# Offspring consume a more obesogenic diet than mothers in response to changing socioeconomic status and urbanization in Cebu, Philippines

**DOI:** 10.1186/1479-5868-6-47

**Published:** 2009-07-27

**Authors:** Anna Kelles, Linda Adair

**Affiliations:** 1 Department of Nutrition, University of North Carolina at Chapel Hill, 123 W. Franklin St. CB#8120, Chapel Hill, NC 27516, USA

## Abstract

**Background:**

Overweight increased among Filipino mothers and offspring from 1994 to 2005 however, a higher rate of increase among mothers resulted in a prevalence 4 times higher than that among offspring in 2005. Our aim was to explore the differential effects of changing income, assets, maternal education, and urbanicity on dietary behaviors of mothers and offspring that may affect overweight risk.

**Methods:**

The study included a cohort of Filipino offspring and their mothers participating in the Cebu Longitudinal Health and Nutrition Survey at four time points from 1994 (n = 1,885 pairs) to 2005 (n = 1,349 pairs). The effect of socioeconomic factors and urbanicity, on dietary behaviors including energy adequacy, percent fat and carbohydrates were examined using longitudinal random-effects regression models.

**Results:**

Mothers and offspring were consistently more likely to consume more calories relative to basal needs as well as a higher percent of calories from fat and a lower percent from carbohydrates with higher socioeconomic status and urbanization. Despite the substantially higher rates of overweight among mothers compared to offspring, offspring consumed a significantly more obesogenic diet than mothers experiencing the same increases in wealth and urbanicity.

**Conclusion:**

Family-based interventions should be developed to counteract the shift towards a more obesogenic diet observed for both Filipino mothers and offspring.

## Introduction

In developing countries undergoing rapid urbanization and social and economic change increases in the consumption of processed foods, animal fats, and simple sugars, as well as an overall increase in total energy intake reflect the nutrition transition [1]. Simultaneously, modernization leads to a shift from active to sedentary occupations, domestic chores, and forms of transportation. These changes in diet and physical activity ultimately lead to the emergence of overweight and obesity in countries with historically high levels of chronic undernutrition [2,3]. Several studies have shown that these trends toward positive energy balance tend to occur first among the wealthy and/or urban subpopulations in developing countries [4-10]. However, there may be substantial heterogeneity in dietary responses to social and economic changes within strata of socioeconomic status or urbanicity. A recent multi-country study found that obesity rates initially increased among adults before children in developing countries [11] provoking another series of important questions. Does this reflect generational or cohort differences? Is it an effect of aging or a differential response to changing environmental and economic circumstances? Answering these questions can help in the development of targeted and effective interventions for overweight populations in a context where chronic undernutrition is still a serious concern.

The Philippines, like many developing countries, has experienced rapid modernization in recent decades. There is also evidence that the nutrition transition has affected this population. A study based on data from the Cebu Longitudinal Health and Nutrition Survey (CLHNS) found that the prevalence of overweight increased significantly among mothers and a cohort of their offspring during a 14 year period [12]. However, in 2005, the prevalence of overweight for mothers (43%) was greater than 4 times that observed for their sons (10%) and daughters (8%) [12]. This suggests that Filipino mothers and offspring may not be responding to the nutrition transition in the same way. Thus, further exploration of these longitudinal data may provide important insights into whether there are generational differences in how key determinants of weight status, namely diet and physical activity, respond to rapidly changing social, economic, and environmental circumstances. One study based on the CLHNS examined moderate to vigorous physical activity patterns and found that occupational, domestic and leisure moderate to vigorous activity decreased from 1998 to 2005 for both mothers and offspring [12]. To examine the other side of the energy balance equation, we use the CLHNS data to explore the differential effects of changing income, assets, maternal education, and urbanicity on three characteristics of maternal and offspring diet that may affect obesity risk: percentage of calories from fat, percentage of calories from carbohydrates and estimated energy adequacy.

## Materials and methods

### Study population

Participants were recruited from metropolitan Cebu, Philippines, which includes Cebu City (the second largest city in the country) and several smaller urban as well as mountainous and coastal rural communities. All pregnant women in 17 urban and 16 rural randomly selected barangays (administrative units) were invited to participate in the study (ages 14.8 to 47.1 years, n = 3,327). Between May 1^st^, 1983 and April 30^th^, 1984 there were 3,080 singleton births identified. Information was collected once during the third trimester of pregnancy, at delivery and bimonthly for 24 months. The survey was extended to include rounds in 1991–2, 1994–5, 1998–2000, 2002, and 2005–6 where the average age of the index offspring were 8.5, 11.5, 15.5, 18.7, and 21.5 years, respectively. For convenience, we refer to these rounds as 1991, 1994, 1998, 2002 and 2005. For each survey year, the analysis samples were restricted to mother/offspring pairs living together in the same household, and included cases where the offspring was a singleton, and neither the mother nor the offspring was pregnant, incapacitated or institutionalized during the given year. Of the pairs who were not included in each survey year, a majority were left out due to the separation of mother and offspring into different households. Maternal education, household income and community urbanicity was lower for these pairs at baseline (1983) than those retained in the study. However, previous studies using the CLHNS show no significant bias due to these differences. Our final sample, comprised of a closed cohort panel, included repeated measurements for mother/offspring pairs in 1994 (n = 1,884), 1998 (n = 1,781), 2002 (n = 1,615), and 2005 (n = 1,349). To ensure comparability across survey years, the 1991 survey was not included in this analysis. Unlike in subsequent surveys, in 1991 mothers were used as a surrogate to recall offspring diet. Prior to the 1994 survey, offspring were too young to have a substantial impact on their dietary choices independent of maternal preferences. The CLHNS protocols were reviewed and approved by the Institutional Review Board of the University of North Carolina at Chapel Hill.

### Measures

#### Dependent variables

Dietary information was collected using 24-hour dietary recalls. One day of intake was recorded from mothers in all years and from offspring in 1994. From 1998 to 2005, two days of dietary recall are available for offspring. Data were collected during in-home interviews performed by highly trained local field staff. All data were checked by editors, and implausible intake values were verified by sending interviewers back to question respondents. To minimize loss of information and maximize the probability of obtaining unbiased estimates [13], offspring intakes are represented by 2-day average intakes for each year from 1998 through 2005. For all survey years, dietary data was collected directly from the offspring with parental supervision.

#### Estimated Energy adequacy (EEA)

Total energy intake (kcal) was calculated using year-appropriate Philippines Food Composition Tables from the Food and Nutrition Research Institute. To create a comparable measure of total energy for mothers and offspring, total intake was divided by BEE to adjust for body size. BEE was estimated using the following most recent WHO/FAO equations based on doubly labeled water studies [14], which account for adolescent energy requirements for growth and development, where age was in years, both height (m) and weight (kg) were measured by trained field staff:

(1) Normal weight mother BEE: (255 - 2.35*age + 361.6*height + 10.12*weight)

(2) Overweight mother BEE: (247 - 2.67*age + 401.5*height + 8.60*weight)

(3) Normal weight daughter BEE: (189 - 17.6*age + 625*height + 7.9*weight)

(4) Overweight daughter BEE: (515.8 - 26.8*age + 347*height + 12.4*weight)

(5) Normal weight son BEE: (68 - 43.3*age + 712*height + 19.2*weight)

(6) Overweight son BEE: (419.9 - 33.5*age + 418.9*height + 16.7*weight)

Height was measured to the nearest 0.1 cm using portable stadiometers and weight was measured to the nearest 0.1 kg using portable scales. Overweight was defined for mothers and children ages 18 and older as a BMI of ≥ 25. For children < 18 years of age (all those in 1994, 1998 and 14 in 2002), the International Obesity Task Force (IOTF) sex and age-specific cut points developed by Cole et al were used to determine overweight [15]. Any change in weight over time for mothers was due primarily to an increase in fat mass. To avoid an artificial increase in energy requirement, maternal weight in 1994 was used to calculate BEE in subsequent years. This method assumes that weight change over time due to changes in lean body mass was negligible. The final variable for mothers and offspring was expressed as a proportion (kcal/BEE) which represents estimated energy adequacy.

#### Diet composition – fat and carbohydrate consumption

Percentage of calories from fat (%FAT) or carbohydrates (%CHO) were calculated by multiplying the total gram intake from the 24-hr recalls by 9 (kcal per 1-unit gram) and 4 respectively and dividing by the total 24-hour kcal intake. Both %FAT and % CHO were represented in the final models as continuous variables.

### Independent variables

#### Maternal education

For each round of the CLHNS, maternal education was recorded as the highest year of education completed. Observations tended to cluster around primary school and secondary school graduation therefore; indicator variables were created to represent: less than primary school graduate, primary school graduate, some high school, and high school graduate and beyond. However, few women attained additional education after the 1983 baseline survey so there is minimal change over time in maternal education.

#### Household income

Total household income included the sum of both cash income from all household members over 6 years of age and the value of in-kind earnings. For comparability over time, income values were deflated to January 1983 values using year-appropriate Philippines consumer price indices from 1994 to 2005. For all analyses, a continuous variable of household income was truncated so that right-skewed outliers were given the value at the 99^th ^percentile of the sample income distribution (n = 21, < 1% of the sample).

#### Household assets

A proxy for wealth was created using household assets represented by the sum of the number of selected possessions ranging from small items such as electric fans to house ownership and construction material. The resulting index took on values from 0 to 11. Previous research has shown that a simple summation of ownership of material goods is an accurate and robust estimate of SES in a developing country context [16].

#### Urbanicity

Recent studies based on the CLHNS have found substantial heterogeneity in the common urban-rural dichotomy [17,18], To minimize misclassification and allow for an exploration of changes in urbanicity over time, an urbanicity index score was assigned to each barangay based on 7 criteria: population size, population density, communication, transportation, healthcare services, education, and market availability [17]. Each category was created with values from 1 to 10 so that a maximum score of 70 represented the most urban community. The urbanicity score was included in our final models as a continuous variable.

#### Household member status

A binary variable indicated whether the participant was a mother (0) or offspring (1). Within each household there was a maximum of 4 observations per household member (one per survey year).

#### Time

Given that the four survey years included in this study were not equally spaced, a series of indicator variables were created to represent 1994, 1998, 2002 and 2005. Significant coefficients on time variables also indicate dietary trends over time.

### Statistical analysis

Descriptive statistics (number of participants, means, standard deviations, and proportions) were used to characterize sample characteristics for each survey year. Our aim was to identify whether change SES and urbanicity over time, had a differential effect on diet patterns of mothers versus offspring. To directly compare the dietary patterns of mothers and offspring over time, separate datasets were created for mothers and offspring, with a maximum of 4 observations per person (representing the 4 survey years). The two datasets were then appended, and a binary indicator household member variable identified the intake value (EEA, %FAT, and %CHO) as belonging to the mother or the offspring within each family. Since the mother-offspring pairs resided in the same household, the value for each independent variable was the same for both household members within each survey year. Therefore, we were able to evaluate possible differences in dietary patterns of mothers versus offspring living in the same environmental conditions.

We used random-intercept mixed models to control for unobserved characteristics associated with clustering of repeat measurement occasions within individuals and for multiple individuals within families. Three separate sets of models were constructed to explore the effect over time of SES and urbanization on EEA, %FAT and %CHO. Our intention was to answer four distinct questions, 1) Do EEA, %FAT and %CHO differ in mothers and offspring residing in the same household? 2) Do changing household income and assets and community level urbanization significantly alter dietary patterns? 3) Is the relationship the same in mothers and their offspring? 4) Do the relationships vary over time? To answer these questions, each model included main effects of member, SES and urbanicity and year, two-way interactions of member with SES and urbanicity and member with time, and 3-way interactions of member, time, and the SES and urbanicity variables.

The coefficients for main effects in each model represented the average effect of SES and urbanicity on maternal diet. Interactions of SES and urbanicity with household member represented the added effect of those variables on the diet of offspring versus mothers. Time interactions tell whether SES and urbanicity affected diet changed over time. Triple interactions of time, member status, and SES and urbanicity tell whether there were differential effects of SES and urbanicity on offspring versus mothers change over time. Preliminary analyses showed a significant interaction between offspring gender and household member status on diet outcomes, therefore analyses were stratified by offspring gender. Results were considered significant at a p value ≤ 0.05 for main effects, and interactions were assessed using partial F tests and considered significant at p ≤ 0.10. To avoid artificially large standard errors of interaction terms due to substantial covariance, backwards deletion was used to eliminate the interaction terms with the time variables that showed minimal impact (T-statistic < |1.0|). All models were adjusted for maternal age using both a continuous and squared age term due to a curvilinear relationship between maternal age and all three diet outcomes. Given that complex longitudinal analyses were performed with multiple time and offspring interactions, the coefficients for the individual interactions are essentially uninterpretable. Therefore, to facilitate the interpretation of the models, we predicted diet outcomes under contrasting circumstances, representing for each year, profiles of high SES and urbanicity (90^th ^percentile of sample level of income, assets and urbanicity and maternal education level = high school graduate+), or low SES and urbanicity (10^th ^percentile of income, assets and urbanicity and maternal education level = primary school graduate). All analyses were performed using Stata 9.2 [19].

## Results

Table 1 presents individual and household characteristics. In general, EEA and %FAT fat increased and %CHO decreased over time among offspring. Among mothers, there were small decreases over time in EEA and %FAT between 1998 and 2005, but %CHO remained relatively constant. Average household assets, income and community urbanicity increased over time but maternal education did not

**Table 1:** Individual, household and community characteristics (Mean ± SD) of mother-offspring pairs from 1994 to 2005 in Cebu, Philippines^a^

Year	**1994**	**1998**	**2002**	**2005**
No. of households	1,884	1,781	1,615	1,311
**Outcome – Dietary characteristics:**				
*Mothers*				
Energy adequacy, (Kcal/BEE)^a^	1.1	1.2	1.1	1.0
Percent calories from CHO^a^, (%)	69.0	68.2	67.8	68.1
Percent calories from fat, (%)	15.8	17.1	16.0	15.7
*Offspring – Females*				
Energy adequacy, (Kcal/BEE)	1.1	1.1	1.3	1.4
Percent calories from CHO, (%)	68.4	64.0	56.0	55.6
Percent calories from fat, (%)	17.2	22.1	26.6	25.8
*Offspring – Males*				
Energy adequacy, (Kcal/BEE)	1.2	1.4	1.5	1.6
Percent calories from CHO, (%)	68.4	64.3	60.5	60.0
Percent calories from fat, (%)	16.9	21.8	22.2	21.6
				
**Individual-level characteristics:**				
*Mothers*				
Age, (years)	38.7 ± 6.1	42.7 ± 6.1	45.9 ± 6.0	48.6 ± 5.9
Education, (years)	7.5 ± 3.9	7.6 ± 3.9	7.6 ± 3.9	7.7 ± 3.9
				
Gender of Offspring (% male)	51.6	53.0	55.7	56.5
*Offspring – No. of Females*	912	837	720	571
Age, (years)	11.5 ± 0.4	14.9 ± 0.4	18.7 ± 0.3	21.5 ± 0.3
Education, (years)	3.7 ± 1.0	7.8 ± 1.5	10.8 ± 2.0	12.1 ± 3.1
				
*Offspring – No. of Males*	968	944	895	740
Age, (years)	11.5 ± 0.4	16.1 ± 0.3	18.7 ± 0.3	21.5 ± 0.3
Education, (years)	3.4 ± 1.2	7.8 ± 1.5	9.5 ± 2.9	10.7 ± 3.9
				
**Household-level characteristics:**				
Household income (pesos/wk)	501.0 ± 407.8	543.3 ± 407.4	578.1 ± 468.8	601.4 ± 555.0
Household assets, (score,1 to 11)	4.0 ± 2.2	4.8 ± 2.2	5.3 ± 2.0	5.5 ± 1.9
				
**Community-level characteristics:**				
Urbanicity (score, 1 to 70)	35.7 ± 13.3	39.0 ± 13.7	41.9 ± 13.8	41.0 ± 13.4

^a^*Notes*: SD = Standard Deviation, Kcal = kilocalories, BEE = Basal Energy Expenditure, CHO = Carbohydrate

Table 2 shows EEA results. Among mothers, a higher EEA was associated with higher education, higher household income and assets, and residence in a more urban community. Maternal EEA declined over time, and the magnitude of the effect of urbanicity on EEA decreased over time. Contrary to our hypothesis, offspring EEA increased more in response to SES and urbanicity, than did maternal EEA. However, the mother-son difference in the effect of urbanicity on EEA decreased over time.

**Table 2:** Longitudinal random-effects regression predicting change in total dietary calories/Basal Energy Expenditure (BEE) for Filipino mothers versus offspring^a^

**Model**	Mother and Daughter	Mother and Son
**Household member:**		
Mother	*Ref^b^*	*Ref*
Offspring	-0.22 (-0.31,-0.13)*** ^c^	-0.13 (-0.26,0.004)^†^
***Independent variables:***		
Maternal education:		
Less than primary school graduate	*Ref*	*Ref*
Primary school graduate	0.07 (0.01,0.12)*	0.11 (0.05,0.17)***
Some high school	0.12 (0.06,0.18)***	0.14 (0.08,0.20)***
High school graduate or higher	0.22 (0.15,0.30)***	0.22 (0.15,0.30)***
Household income, (per 100 pesos)	0.006 (-0.001,0.013)	0.008 (0.003,0.01)***
Household assets, (score,1 to 11)	0.01 (-0.004,0.02)	0.01 (-0.001,0.03)^†^
Urbanicity, (score, 1 to 70)	0.006 (0.004,0.008)***	0.006 (0.003,0.008)***
Time:		
1994	*Ref*	*Ref*
1998	-0.001 (-0.10,0.09)	0.27 (0.13,0.40)***
2002	0.04 (-0.07,0.15)	0.04 (-0.10,0.19)
2005	-0.20 (-0.33,-0.07)**	0.01 (-0.14,0.17)
***Offspring – independent variable interactions:***		
Offspring*Primary school graduate	-0.04 (-0.11,0.03)	-0.06 (-0.14,0.02)
Offspring*Some high school	0.01 (-0.06,0.08)	-0.004 (-0.08,0.07)
Offspring*High school graduate or higher	-0.03 (-0.12,0.06)	-0.03 (-0.12,0.07)
Offspring*Household income	-0.004 (-0.01,0.002)	-0.002 (-0.008,0.004)
Offspring* Household assets	0.03 (0.01,0.04)***	0.01 (-0.001,0.03)^†^
Offspring*Urbanicity	0.003 (0.001,0.005)*	0.005 (0.001,0.008)**
***Independent variable – time interactions:***		
Offspring*1998	0.05 (-0.006,0.10)^†^	0.16 (-0.02,0.34)^†^
Offspring*2002	0.24 (0.18,0.30)***	0.49 (0.31,0.68)***
Offspring*2005	0.39 (0.33,0.46)***	0.92 (0.73,1.12)***
Household income*1998	0.008 (-0.01,0.02)^†^	---------------
Household income*2002	-0.003 (-0.01,0.01)	---------------
Household income*2005	-0.005 (-0.01,0.004)	---------------
Household assets*1998	-0.006 (-0.02,0.01)	-0.002 (-0.02,0.01)
Household assets*2002	-0.008 (-0.02,0.01)	-0.003 (-0.02,0.01)
Household assets*2005	0.02 (0.01,0.05)*	-0.02 (-0.04,-0.01)**
Urbanicity*1998	-0.003 (-0.005,-0.001)*	-0.005 (-0.01,-0.002)**
Urbanicity*2002	-0.002 (-0.005,-0.001)*	-0.002 (-0.005,0.001)
Urbanicity*2005	-0.002 (-0.005,0.000)^†^	-0.001 (-0.005,0.002)
***Triple interactions:***		
Offspring*Urbanicity*1998	---------------	-0.002 (-0.006,0.002)
Offspring*Urbanicity*2002	---------------	-0.005 (-0.009–0.001)*
Offspring*Urbanicity*2005	---------------	-0.009 (-0.01,-0.004)***

^a^All models are adjusted for maternal age

^b^*Notes*: Ref = referent category

^c†^P < 0.10, *P < 0.05, **P < 0.01, ***P < 0.0001

Among mothers, a higher %FAT (Table 3) and lower %CHO (Table 4) was associated with higher education, household income and assets, and residence in a more urban environment. As indicated by the consistently significant coefficients on terms representing household member interactions with income and maternal education, offspring increases in %FAT and decreases in %CHO were larger than their mothers' in response to the common household environment. The magnitude of the maternal-offspring difference in %FAT and %CHO increased over time. This difference was further magnified between mothers and daughters by increases in household assets (for %FAT) and between mothers and sons with increases in urbanicity (calories from carbohydrates).

**Table 3:** Longitudinal random-effects regression predicting percent of dietary calories from fat for Filipino mothers versus offspring^a^

**Model**	Mother and Daughter	Mother and Son
**Household member:**		
Mother	*Ref^b^*	*Ref*
Offspring	3.40 (1.01,5.79)** ^c^	0.75 (-1.10,2.59)
***Independent variables:***		
Maternal education:		
Less than primary school graduate	*Ref*	*Ref*
Primary school graduate	1.46 (0.25,2.67)*	2.04 (0.84,3.25)**
Some high school	3.77 (2.53,5.01)***	3.32 (2.15,4.49)***
High school graduate or higher	6.22 (4.62,7.83)***	6.95 (4.52,8.47)***
Household income, (per 100 pesos)	0.29 (0.12,0.46)**	0.17 (0.08,0.26)***
Household assets, (score,1 to 11)	1.10 (0.72,1.049)***	0.74 (0.51,0.97)***
Urbanicity, (score, 1 to 70)	0.08 (0.05,0.12)***	0.14 (0.09,0.18)***
Time:		
1994	*Ref*	*Ref*
1998	0.34 (-1.86,2.54)	4.88 (2.80,6.94)***
2002	0.72 (-1.98,3.41)	2.69 (0.48,4.90)*
2005	1.60 (-1.73,4.92)	0.23 (-2.15,2.61)
***Offspring – independent variable interactions:***		
Offspring*Primary school graduate	-0.40 (-1.87,1.06)	-1.13 (-2.60,0.33)
Offspring*Some high school	-0.86 (-2.35,0.64)	-1.33 (-2.75,0.08)^†^
Offspring*High school graduate or higher	-1.23 (-3.18,0.72)	-2.63 (-4.51,-0.75)**
Offspring*Household income	-0.19 (-0.34,-0.05)**	-0.08 (-0.21,0.04)
Offspring*Household assets	-0.31 (-0.80,0.17)	0.17 (-0.14,0.47)
Offspring*Urbanicity	0.02 (-0.02,0.06)	0.03 (-0.02,0.07)
***Independent variable – time interactions:***		
Offspring*1998	1.76 (-1.30,4.82)	2.50 (1.17,3.84)***
Offspring*2002	6.40 (2.73,10.08)**	4.90 (3.51,6.30)***
Offspring*2005	2.92 (-1.56,7.39)	5.37 (3.63,6.57)***
Household income*1998	0.17 (-0.04,0.39)	---------------
Household income*2002	0.01 (-0.20,0.22)	---------------
Household income*2005	-0.17 (-0.38,0.03)^†^	---------------
Household assets*1998	-0.48 (-0.99,0.03)^†^	---------------
Household assets*2002	-0.50 (-1.04,0.05)^†^	---------------
Household assets*2005	-0.41 (-1.03,0.22)	---------------
Urbanicity*1998	---------------	-0.08 (-0.13,-0.04)**
Urbanicity*2002	---------------	-0.09 (-0.14,-0.04)**
Urbanicity*2005	---------------	-0.05 (-0.10,0.01)^†^
***Triple interactions:***		
Offspring*Household assets*1998	0.81 (0.17,1.45)*	---------------
Offspring*Household assets*2002	0.57 (-0.13,1.27)	---------------
Offspring*Household assets*2005	0.98 (0.16,1.79)*	---------------

^a^All models are adjusted for maternal age

^b^*Notes*: Ref = referent category

^c†^P < 0.10, *P < 0.05, **P < 0.01, ***P < 0.0001

**Table 4:** Longitudinal random-effects regression predicting dietary calories from carbohydrates for Filipino mothers versus offspring^a^

**Model**	Mother and Daughter	Mother and Son
**Household member:**		
Mother	*Ref^b^*	*Ref*
Offspring	-2.65 (-4.70,0.61)* ^c^	-2.24 (-3.57,-0.91)**
***Independent variables:***		
Maternal education:		
Less than primary school graduate	*Ref*	*Ref*
Primary school graduate	-1.23 (-2.53,0.07)^†^	-1.87 (-3.16,-0.59)**
Some high school	-3.71 (-5.04,-2.38)***	-3.06 (-4.32,-1.81)***
High school graduate or higher	-6.28 (-8.00,-4.56)***	-7.16 (-8.78,-5.53)***
Household income, (per 100 pesos)	-0.29 (-0.46,-0.13)***	-0.22 (-0.32,-0.12)***
Household assets, (score,1 to 11)	-0.96 (-1.23,-0.70)***	-0.93 (-1.18,-0.68)***
Urbanicity, (score, 1 to 70)	-0.09 (-0.12,-0.05)***	-0.10 (-0.15,-0.05)***
Time:		
1994	*Ref*	*Ref*
1998	2.36 (-0.90,3.83)**	-2.36 (-5.18,-0.81)**
2002	1.17 (-0.47,2.80)	-1.87 (-4.20,0.45)
2005	-0.01 (-1.80,1.77)	1.14 (-1.36,3.64)
***Offspring – independent variable interactions:***		
Offspring*Primary school graduate	0.25 (-1.32,1.81)	1.06 (-0.53,2.64)
Offspring*Some high school	1.37 (0.22,2.97)^†^	1.56 (0.03,2.97)*
Offspring*High school graduate+	1.62 (-0.47,3.71)	2.77 (0.74,4.71)**
Offspring*Household income	0.23 (0.08,0.38)**	0.12 (-0.01,0.25)^†^
Offspring*Household assets	-0.01 (-0.35,0.32)	-0.03 (-0.36,0.29)
Offspring*Urbanicity	-0.01 (-0.06,0.03)	-0.01 (-0.06,0.03)
***Independent variable – time interactions:***		
Offspring*1998	-4.98 (-6.46,-3.51)***	-2.38 (-3.78,-0.97)**
Offspring*2002	-11.07 (-12.66,-9.48)***	-6.68 (-8.15,-5.22)***
Offspring*2005	-10.95 (-12.66,-9.25)***	-8.10 (-9.64,-6.56)***
Household income*1998	-0.23 (-0.42,-0.04)*	---------------
Household income*2002	-0.09 (-0.28,0.10)	---------------
Household income*2005	0.07 (-0.12,0.26)	---------------
Urbanicity*1998	---------------	0.05 (-0.002,0.10)^†^
Urbanicity*2002	---------------	0.04 (-0.01,0.09)
Urbanicity*2005	---------------	-0.01 (-0.07,0.04)

^a^All models are adjusted for maternal age

^b^Ref = referent category

^c†^P < 0.10, *P < 0.05, **P < 0.01, ***P < 0.0001

**Figure 1:**
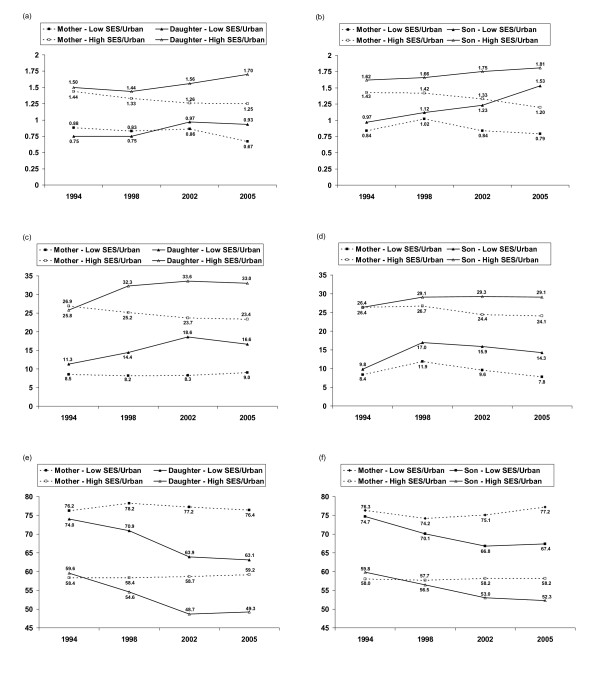
**Predicted prevalence of (a) total calories as a proportion of basal energy expenditure for mothers versus daughters in both a high and Low SES-urbanicity environment, (b) total calories as a proportion of basal energy expenditure for mothers versus sons in both a high and Low SES-urbanicity environment, (c) proportion of total calories from fat for mothers versus daughters in both a high and Low SES- urbanicity environment, (d) proportion of total calories from fat for mothers versus sons in both a high and Low SES-urbanicity environment, (e) proportion of total calories from carbohydrates for mothers versus daughters in both a high and Low SES- urbanicity environment, (f) proportion of total calories from carbohydrates for mothers versus sons in both a high and Low SES-urbanicity environment**. As noted in the text, a high SES-urbanicity environment is represented jointly by the 90^th ^percentile of income, assets and urbanicity and maternal education level = high school graduate or higher, and low SES-urbanicity is represented jointly by the 10^th ^percentile of income, assets and urbanicity and maternal education level = primary school graduate.

Figure 1 presents the predicted dietary trends over time for Filipino mothers compared to their offspring in contrasting high and low SES and urbanicity environments. Mothers and offspring from high SES urban households consume substantially more kilocalories relative to basal needs as well as a higher %FAT and lower %CHO. However, predicted EEA and %FAT decreased over time for mothers but increased over time for offspring, while predicted %CHO remained relatively constant for mothers but decreased substantially for offspring. Over time, the divergence in EEA between mothers and sons was greater than the divergence observed between mothers and daughters. In contrast, the divergence in %FAT and in %CHO between mothers and daughters was greater than the divergence observed between mothers and sons.

## Discussion

Our objective was to explore the differential effect of changes in socioeconomic status and urbanization on the diet patterns of Filipino mothers compared to their offspring. We found with increasing socioeconomic status and urbanization, mothers and offspring were consistently more likely to consume more calories relative to basal needs as well as a higher percent of calories from fat and a lower percent from carbohydrates. This is consistent with dietary changes observed in low-income developing countries experiencing rapid modernization [20-23]. In light of previous developing country research documenting a transition to overweight first among adults leading to a dual-burden of overweight among adult and with underweight among offspring, we hypothesized that dietary trends among Filipino mothers would become more obesogenic in response to urbanization and improvements in SES [11,24,25]. Contrary to our hypothesis, offspring consumed a diet with higher EEA, a higher %FAT and a lower %CHO than their mothers experiencing the same increases in wealth and urbanicity. Our findings may differ from those in previous studies because of our focus on adolescent offspring rather than young children [25-29]. Unlike infants and children whose diets are heavily determined by mothers or caretakers, adolescents may be more responsive to a changing food environment associated with modernization owing to a greater exposure to different food environments outside the home (e.g. work, school and after school social settings).

While at present, the Filipino young adults in the CLHNS have a substantially lower obesity prevalence than their mothers, previous research suggests that they may in fact be at an increased risk for substantial increases in overweight as they move into middle adulthood. Using the CLHNS, we observed a modest but steady increase in overweight and a decrease in underweight among Filipino offspring from 1991 to 2005 [30]. In addition, we observed a steady drop in the number of offspring participating in physical activity, particularly among the wealthy urban subpopulation [30]. Unless interventions are designed to address these obesogenic behavior trends, overweight may eventually exceed the high levels currently observed in their mother's generation. Since overweight and its associated diseases are already a serious public health problem among Filipino adults [31,32], interventions should be developed at the family level. These interventions should focus on a healthy balanced diet versus calorie restriction given that dual-burden households with an overweight mother and underweight offspring tend to exist in wealthy urban environments where we expect to observe continual increases in offspring overweight [30].

This study has a number of limitations, primarily concerning the method for creating the energy adequacy outcome. Use of BEE in lieu of total energy expenditure (TEE) may have biased our estimates. We did not have data to estimate physical activity duration and intensity and therefore could not accurately estimate energy expenditure from physical activity. Previous findings using the CLHNS data show that a greater number of sons participate in occupational and leisure moderate/vigorous activity than mothers and daughters suggesting that use of BEE may have inflated the extent to which energy need was exceeded in this study, particularly for sons.

Our results show a decrease in EEA over time for mothers despite a continued increase in overweight. This brings in to question our method for estimating EEA for mothers. We assumed that weight changes from 1994–2005 primarily reflected increases in fat mass rather than fat free mass, particularly in light of our observation that occupational and leisure time physical activity declined during this period [30]. Since an increase in fat mass would not increase energy need, we used mothers' weight from 1994 to estimate BEE for subsequent years. The assumption that fat free mass remained unchanged may not hold for older women who experienced age-related losses of fat free mass [33]. However, the inclusion of maternal age in the models used to estimate EEA reduced the likelihood that our estimates are biased for older women. According to the Institute of Medicine [14], different equations are necessary to calculate BEE for overweight versus normal or underweight individuals. We did this for women based on their weight status in 1994. However, we used their 1994 weight status to estimate energy adequacy in subsequent years under the assumption that subsequent weight changes would primarily reflect changes in fat mass which would not alter estimated BEE. Moreover, the equations were designed to measure BEE across a population of individuals with varying weights and not necessarily for individuals with changing weight over time. It is unlikely that there was a substantial shift in energy need for mothers as they passed the BMI = 25 kg/m^2 ^threshold which defines overweight. In parallel with the decline in EEA, we observed a decrease over time in %FAT, which is not based on weight status. The apparent paradox of increased body weight with decreased EEA over time may be explained by a disproportionately greater decrease in energy expended in physical activity compared to energy intake which could still result in continued weight gain over time.

In our study, BEE was calculated using equations based on data from an American population. Previous studies have found that Asians tend to have higher fat mass and less fat free mass compared to Americans with the same BMI; therefore the estimation equations may have overestimated actual BEE (fat mass is less metabolically active than fat free mass). However, since our population is ethnically homogenous the extent to which BEE may be overestimated is probably consistent across the sample and therefore we do not expect significant differential misclassification in the ranking of individuals by BEE levels.

As with all longitudinal studies, there is always the possibility of selection bias due to attrition. Previous studies using the CLHNS have found no significant affect of selection bias due to attrition; however attrition may have reduced the generalizability to the original source population. Another possible limitation of this study was the assumption that the accuracy of dietary intake reports was the same for mothers and offspring. In developed countries, studies have found that overweight and obese individuals are more likely to underreport calorie and fat intakes possibly in response to social pressures to be thin [34-38]. However, given that mothers and offspring consume a more obesogenic diet in response to increased wealth and urbanicity, non-random misreporting of dietary behaviors between mothers and offspring is unlikely. A final potential limitation of this study is the possibility that income is endogenous to the specified models. It may be that there are unobservable variables that are associated with both income and eating behaviors for mothers and offspring. This would be of particular concern if we expected that the nature of this association differed for mothers compared to their offspring. We have no reason to believe that this is the case.

There are several unique strengths of this study. Previous research has documented dietary behavior changes in developing countries such as increased total energy intake from processed foods high in sugar and fat intake from animal sources. However no studies to date have explored a possible differential response in dietary behaviors of adults and offspring to changing SES and urbanization that might explain discrepant weight status. Because of the detailed environmental, socioeconomic, and demographic information of the CLHNS from individual, household and community-levels, we had the unique opportunity in this study to explore intergenerational responses to changing community and household level conditions indicative of modernization. Additionally, the wealth of data of the CLHNS allowed for a detailed exploration of multiple dimensions of diet. Finally, this study documents dietary trends through important period of development from adolescence to adulthood for offspring.

## Conclusion

Several studies have identified the coexistence of overweight and underweight in developing countries [25,27,29]. However, few studies have explored the possible differential effect of SES and urbanization on weight-related behavior patterns that might lead to this dual-burden phenomenon. Given that the overweight individual is commonly an adult and the underweight individual an offspring in these dual-burden pairs, it is a logical assumption to believe that there is a generational difference in behavioral responses to changing social and economic factors. With modernization comes increased access to western dietary and body image ideals, especially among the urban wealthy. Younger generations may be more susceptible to these influences. Our findings suggest that Filipino offspring are as likely, if not more likely, as their mothers to adopt an obesogenic lifestyle in response to increased wealth and urbanicity. Given the evidence from this study, it may be prudent for Filipino public health officials to prepare for a continued shift towards an obesogenic lifestyle and a likely increase in the overweight trend among the urban wealthy.

## Competing interests

The authors declare that they have no competing interests.

## Authors' contributions

AJ conceived the study hypothesis, conducted data analyses and wrote the manuscript. LA was responsible for data management of the Cebu Longitudinal Health and Nutrition Survey and contributed to data analyses and the writing of the manuscript.
